# Using geometric algebra to represent curvature in shell theory with applications to Starling resistors

**DOI:** 10.1098/rsos.171212

**Published:** 2017-11-29

**Authors:** A. L. Gregory, A. Agarwal, J. Lasenby

**Affiliations:** Department of Engineering, Cambridge University, Trumpington Street, Cambridge, CB2 1PZ, UK

**Keywords:** Starling resistor, geometric algebra, shell theory

## Abstract

We present a novel application of rotors in geometric algebra to represent the change of curvature tensor that is used in shell theory as part of the constitutive law. We introduce a new decomposition of the change of curvature tensor, which has explicit terms for changes of curvature due to initial curvature combined with strain, and changes in rotation over the surface. We use this decomposition to perform a scaling analysis of the relative importance of bending and stretching in flexible tubes undergoing self-excited oscillations. These oscillations have relevance to the lung, in which it is believed that they are responsible for wheezing. The new analysis is necessitated by the fact that the working fluid is air, compared to water in most previous work. We use stereographic imaging to empirically measure the relative importance of bending and stretching energy in observed self-excited oscillations. This enables us to validate our scaling analysis. We show that bending energy is dominated by stretching energy, and the scaling analysis makes clear that this will remain true for tubes in the airways of the lung.

## 1. Introduction

Self-excited oscillations of flexible tubes driven by fluid flow have been a subject of interest for some time, and there is a considerable literature on the subject, which is reviewed in [1–6]. Experimental rigs designed to study this phenomenon are often called Starling resistors. We are interested in this phenomenon because of its possible relevance to wheezing in the lung [7], which is one of the most commonly heard lung sounds used for diagnosis [8,9]. Previous work on Starling resistors has largely used water as the working fluid. In the lung, the working fluid is air. This means that the density ratio between the working fluid and the tube material in the lung is significantly different from almost all of previously completed work on Starling resistors. Previous modelling work usually neglects wall inertia, using instead a ‘tube law’ [10], but there is strong evidence that wall inertia is significant in the lung from [8,11], where it was shown that when the density of the fluid breathed in is changed, the frequency of the wheezes is not affected significantly. It is clear, therefore, that the change in density ratio results in a qualitatively different mechanism. For this reason, we have been conducting our own experiments, and creating models to understand the onset of oscillations.

The flexible tube itself is generally modelled as an elastic shell. Traditional shell theories [12–17] are well developed but difficult to implement. We have found that linearized shell theories do not provide good predictions of the frequencies of oscillation, and we believe that this is due in part to the fact that we have observed that oscillations start from a collapsed or partially collapsed state. To use current geometrically nonlinear shell theories would require a numerical simulation of a very complex fluid structure interaction problem, which would be of similar value to experimental results (though arguably harder to implement), and would provide the same problem of being difficult to physically interpret due to the complex nature of the oscillations observed. Instead, we would like to gain a physical understanding of the important mechanisms behind the oscillations, and this is difficult with shell theories based in differential geometry [14,15], in particular, due to the lack of physical interpretations of the change of curvature tensor in general situations. We recently introduced geometric algebra to shell theory [18], which allowed us to express the fundamental laws in a component-free form and clarify the role of angular velocity and moments through the use of bivector representation. For an introduction to the basics of geometric algebra see [19]. One of the most powerful aspects of geometric algebra lies in the use of rotors to represent rotations. In [20], these have been used to simplify Simo and Vu Quoc’s numerical algorithm [21] for modelling the nonlinear behaviour of rods. In projective and conformal geometry [19], §10 rotors have allowed geometric primitives to be represented in a more simple and lucid manner, and in relativity [22] and relativistic analogies [23] rotors can simplify transformation between frames of reference. In this paper, we make use of rotors to better understand the change of curvature of a shell, which is of prime importance to the constitutive law of the shell, but whose representation has long caused controversy. In [12] at least 10 different linearized shell theories are presented, and the differences are primarily caused by disagreements over how to represent changes of curvature. The author in [14] has provided a tensor definition of the change of curvature that has become accepted; however, the utility of this expression is limited by its complexity. We have been able to simplify the representation of this tensor using rotors, allowing a more lucid and physical interpretation of changes of curvature. We take advantage of this to allow us to understand the importance of the change of curvature in the context of our Starling resistor experiments.

To compare results from the shell theory to our experimental results, we need to be able to calculate the kinematic parameters associated with the deformation of the flexible tube. To enable this, we use stereoscopic imaging, which, to our knowledge, is the first use in the study of Starling resistors. We take high-speed video of the tube at the onset of oscillation, and are able to track the motion of the surface, and consequently compare the predictions of shell theory with empirical calculation.

## 2. Understanding changes in curvature

There is an energy associated with any deformation of a shell, and Koiter [17] proposes the following form for this energy:

2.1 $$\rho_{0}U=\frac{Eh}{2(1-\nu^{2})}((1-\nu )\mathrm{tr}(E^{2})+\nu \mathrm{tr}{\left(E\right)}^{2})+\frac{Eh^{3}}{24(1-\nu^{2})}((1-\nu )\mathrm{tr}(H^{2})+\nu \mathrm{tr}{\left(H\right)}^{2}).$$

Here, *U* is the internal energy per unit mass of the shell, defined on the reference configuration, *ρ*_0_ is the time-independent area density of the shell on the reference configuration, *E* is Young’s modulus, *ν* is Poisson’s ratio, *h* is the shell thickness (which in Koiter’s theory is assumed constant), E is the two-dimensional Green–Lagrange strain tensor defined on the reference configuration, H is the change of curvature tensor and tr is the trace operator. From (2.1), we can derive the governing equations of the shell (for more details see [18]). The first term on the right-hand side of (2.1) represents the stretching energy, and the second term represents the bending energy.

In general, a shell is a body in which the thickness is smaller than the other relevant defining length scales. The use of (2.1) for the energy of deformation implies that we additionally assume that the mid-surface of the body remains the mid-surface under deformation, a material line that is normal to the mid-surface remains normal to it under deformation, the shell thickness remains constant with time, the first and second moments of density relative to the mid-surface are zero, and strains within the shell are small and so is the normal stress (see [18] for further discussion).

Following the notation of [18], we take *B* and *S* to be the reference and spatial configurations of the shell, and *X*∈*B* and *x*∈*S* to be locations on these configurations. By *ϕ*_*t*_ we denote the motion of the shell, meaning that, at time *t*, the point *X*∈*B* is at the position *ϕ*_*t*_(*X*)∈*S*; G and g are the identity functions on the reference and spatial configurations. *Y* and *y* are vectors within the tangent spaces of *B* and *S*, respectively; {*X*^*i*^},*i*=1,2 is a coordinate system on the reference configuration *B*, which we can then use to define the frame on the reference configuration {*E*_*i*_=∂*X*/∂*X*^*i*^}. By {*E*^*i*^} we denote the reciprocal frame that satisfies $E^{i}\cdot E_{j}=\delta_{j}^{i}$, and {*x*^*i*^}, {*e*_*i*_} and {*e*^*i*^} are the similarly defined coordinate system, frame and reciprocal frame on the spatial configuration, respectively. The shell undergoing deformation is embedded within a flat three-dimensional Euclidean space $E^{3}$.

If *A* and *B* are general multivectors, then *AB* is the geometric product between them, *A*⋅*B* is the inner product, *A*∧*B* is the outer product and *A*×*B* is the commutator product, defined by $A\times B=\frac{1}{2}(AB-BA)$ (see [19], §4.1.3). We also take **×** (compared to ×) to be the cross product between two vectors. If *I* is the pseudoscalar of a three-dimensional space, and *a* and *b* are vectors, then *a***×***b*=−*Ia*∧*b*.

We are particularly interested in the change of curvature that is encoded in H. To understand this, we must understand the curvature tensors on the reference and spatial configurations B and b. If *E*_3_ and *e*_3_ are the normal vectors to the reference and spatial configurations, respectively, then B(*Y*) and b(*y*) are given by

2.2 $$B(Y)=-Y\cdot \partial E_{3}\;\mathrm{and}\;b(y)=-y\cdot \partial e_{3},$$

where ∂ is the intrinsic vector derivative to any surface. The relationship between ∂ and the vector derivative of $E^{3}$ is explained in [18]. On *B* we can expand ∂ as ∂=*E*^*i*^∂/∂*X*^*i*^ and on *S* we can expand it as ∂=*e*^*i*^∂/∂*x*^*i*^ [19], §6.5.1. We see that B and b give non-zero results if the surface is not flat. The change of curvature tensor H is given by

2.3 $$H(Y)=\bar{F}bF(Y)-B(Y),$$

where F is the deformation gradient, defined by F(*Y*)=*Y* ⋅∂*ϕ*_*t*_(*X*). We see that F maps from the tangent space of *B* to the tangent space of *S*, providing information about the local deformation of the surface, and $\bar{F}$ is the adjoint of F, i.e. $F(Y)\cdot y=Y\cdot \bar{F}(y)$. The strain tensor E, used in the constitutive law (2.1), is given by

2.4 $$E(Y)=\frac{1}{2}(\bar{F}F(Y)-Y).$$

This much is well known, though in other treatments coordinate-dependent definitions of H are used (e.g. in [14]). To make further progress, we will now use rotors to better understand what will produce changes in H.

To begin, we note that we can perform a polar decomposition on F such that F(*Y*)=RU(*Y*), where $\bar{R}=R^{-1}$, detR=1 and $\bar{U}=U$. We see that R encodes rotation, and U encodes stretching. We can choose to consider a frame {*E*_*i*_} on the reference configuration that is locally orthonormal.^1^ In this case, *E*_3_=*E*_1_**×***E*_2_=−*I*_3_(*E*_1_∧*E*_2_)=−*I*_3_(*E*_1_*E*_2_), where *I*_3_ is the pseudoscalar of three-dimensional Euclidean space $E^{3}$.

To find *e*_3_, we need two unit vectors in the tangent space of the spatial configuration that are oriented in the same way as the pair F(*E*_1_),F(*E*_2_), and are orthonormal. This pair of unit vectors is given by R(*E*_1_),R(*E*_2_) and so *e*_3_ is given by *e*_3_=R(*E*_1_)**×**R(*E*_2_)=−*I*_3_(R(*E*_1_)∧R(*E*_2_))=−*I*_3_(R(*E*_1_)R(*E*_2_)). The fact that the function R is a rotation means that it has an associated rotor *R* such that $R(Y)=RY\tilde{R}$, where *R* is an even multivector that satisfies $\tilde{R}R=R\tilde{R}=1$, and $\tilde{R}$ is the reverse of *R*. This allows us to write *e*_3_ as

2.5 $$e_{3}=-I_{3}(\left(RE_{1}\tilde{R})(RE_{2}\tilde{R}\right))=-I_{3}(R(E_{1}E_{2})\tilde{R})=RE_{3}\tilde{R},$$

where we have used the fact that any rotor will commute with *I*_3_. Thus we have shown that the rotation associated with the deformation is also the rotation between the normal vectors *E*_3_ and *e*_3_, which makes intuitive sense. We have also extended the range and domain of R to $E^{3}$, while the range and domain of U is still constrained to the tangent space of the reference configuration, and the range and domain of F are constrained to the tangent spaces of the spatial and reference configuration, respectively.

Two results that we will find useful are

2.6a $$Y\cdot \partial \tilde{R}=-\tilde{R}(Y\cdot \partial R)\tilde{R}$$

and

2.6b $$F(Y)\cdot \partial e_{3}=Y\cdot \partial e_{3}.$$

We see that (2.6a) follows from $\tilde{R}R=1$; (2.6b) has implicit assumptions that require explanation. The expression on the left of (2.6b) tells us how *e*_3_ varies over the spatial configuration in the direction defined by F(*Y*), which lies in the tangent space of the spatial configuration. On the right of (2.6b), *e*_3_=*e*_3_(*x*) has been mapped to a vector field on the reference configuration such that *e*_3_(*X*)=*e*_3_(*ϕ*_*t*_(*X*))∀*X*∈*B*. This allows the expression on the right to tell us how *e*_3_ varies over the reference configuration in the direction defined by *Y* , which is tangent to the reference configuration. The equality of these expressions is a standard result when mapping derivatives between manifolds, which can be proved by considering derivatives with respect to convected coordinates that satisfy $x^{i}(x)=X^{i}(\phi_{t}^{-1}(x))$. From this point we will assume that {*x*^*i*^} are convected coordinates.

Using these results, we can write $\bar{F}bF(Y)$ as $\bar{F}bF(Y)=-\bar{F}(Y\cdot \partial e_{3})$, and the argument of $\bar{F}$ can be expressed as

2.7 $$\begin{array}{rl} Y\cdot \partial e_{3}=Y\cdot \partial (RE_{3}\tilde{R}) & =(Y\cdot \partial R)E_{3}\tilde{R}+R(Y\cdot \partial E_{3})\tilde{R}+RE_{3}(-\tilde{R}(Y\cdot \partial R)\tilde{R})\\ & =R(Y\cdot \partial E_{3})\tilde{R}+[(Y\cdot \partial R)\tilde{R}]RE_{3}\tilde{R}-RE_{3}\tilde{R}[(Y\cdot \partial R)\tilde{R}]\\ & =R(Y\cdot \partial E_{3})\tilde{R}+[(Y\cdot \partial R)\tilde{R}]e_{3}-e_{3}[(Y\cdot \partial R)\tilde{R}]\\ & =R(Y\cdot \partial E_{3})\tilde{R}+[2(Y\cdot \partial R)\tilde{R}]\times e_{3}\\ & =R(Y\cdot \partial E_{3})+[2(Y\cdot \partial R)\tilde{R}]\times e_{3}, \end{array}$$

where × is the commutator product. Hence, we can express $\bar{F}bF(Y)$ as

2.8 $$\begin{array}{rl} \bar{F}bF(Y) & =-\bar{F}R(Y\cdot \partial E_{3})-\bar{F}([2(Y\cdot \partial R)\tilde{R}]\times e_{3})\\ & =-U(Y\cdot \partial E_{3})-\bar{F}([2(Y\cdot \partial R)\tilde{R}]\times e_{3})\\ & =UB(Y)+\bar{F}(e_{3}\times [2(Y\cdot \partial R)\tilde{R}]), \end{array}$$

and finally we obtain an expression for H,

2.9 $$\begin{array}{rl} H(Y) & =(U-G)B(Y)+\bar{F}(e_{3}\times [2(Y\cdot \partial R)\tilde{R}])\\ & =(U-G)B(Y)+\bar{F}(\left[RE_{3}\tilde{R}]\times [2(Y\cdot \partial R)\tilde{R}\right]). \end{array}$$

This shows that there are two contributions to H. Firstly, if the reference configuration is at all curved (i.e. B(*Y*) is non-zero), then the strain of the shell, encoded in U−G, will result in a change of curvature. The second contribution is due to variation of the rotor *R* over the shell. These two kinds of change of curvature are illustrated well by an inflating sphere and deformation of a flat plate. As a sphere is inflated to become a larger sphere, the normal vector is unchanged, i.e. *e*_3_=*E*_3_, hence *R*=1 everywhere. This means that the second term in our expression for H will be zero. However, the surface of the sphere will stretch, meaning that U−G will be non-zero. In addition, B will be non-zero for a sphere, which tells us that the first term in our expression for H will be non-zero. By contrast, for a flat plate B(*Y*) will be zero, meaning that *R* must vary over the plate in order for there to be any change of curvature.

A variant on this expression for H can be obtained if we express the rotor *R* as $R=\exp(-A/2)=\exp(-\hat{A}\theta /2)$, where *A* is a bivector aligned with the plane of rotation whose magnitude is equal to the angle of rotation *θ* and $\hat{A}$ is a unit bivector (${\hat{A}}^{2}=-1$). Note that the direction of rotation is defined by the sign of *θ* and the orientation of $\hat{A}$ together. We can set the convention that *θ*≥0, in which case *θ* and $\hat{A}$ are uniquely defined if *A* is known. Given this definition, we can express *Y* ⋅∂*R* as,

2.10 $$Y\cdot \partial R=-\frac{Y\cdot \partial A}{2}\exp\left(-\frac{A}{2}\right)=-\frac{Y\cdot \partial A}{2}R.$$

Using this, we can express H as

2.11 $$H(Y)=(U-G)B(Y)-\bar{F}(\left(RE_{3}\tilde{R})\times (Y\cdot \partial A\right)).$$

The term that $\bar{F}$ operates on is the commutator product of a vector and bivector, so we can replace the commutator product with a dot product,

2.12 $$H(Y)=(U-G)B(Y)-\bar{F}(\left(RE_{3}\tilde{R})\cdot (Y\cdot \partial A\right)).$$

Taking the inner product of a bivector with $RE_{3}\tilde{R}=e_{3}$ means that only vectors tangential to the spatial configuration are retained, which then means that $\bar{F}$ can operate and return vectors tangential to the reference configuration. Hence, our description confirms that the range and domain of H are both the tangent space of the reference configuration.

The explicit expression for the two possible contributions to change of curvature, shown in (2.12), gives a new decomposition of the change of curvature tensor which will be of use when we try to understand the importance of bending in the Starling resistor.

## 3. Experiment description

### 3.1. Experimental set-up

**Figure 1.**
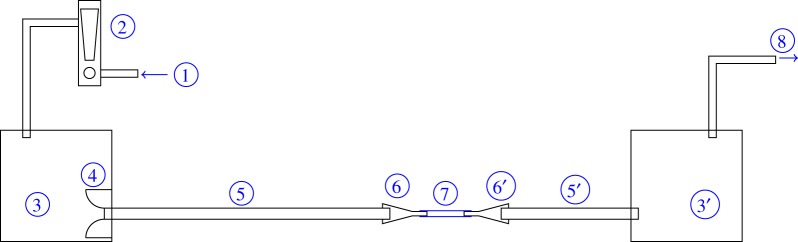
Schematic of Starling resistor experiment. (1) Flow inlet, (2) rotameter, (3/3′) settling chambers, (4) clean flow inlet, (5/5′) clean flow tubes, (6/6′) contraction and expansion, (7) flexible tube and (8) tube to suction fan. The downstream settling chamber is approximately 4 *m*^3^, while the upstream settling chamber is 0.03 *m*^3^.

Figure 1 shows a schematic of the experimental set-up used to investigate the oscillations of flexible tubes. Air flows into the system through (1), and then through a rotameter (2) used to monitor flowrate. The noise that the rotameter introduces into the flow, and any other noise, is isolated from the flexible tube by the upstream settling chamber (3). Air flows into the upstream clean flow tube (5) section via a shaped inlet (4) that reduces separation. A contraction (6) leads to the flexible tube (7), before an expansion (6′) leads to the downstream clean flow tube (5′) that exits into the downstream settling chamber (3′). Suction is provided by a fan (8). The downstream settling chamber (3′) isolates the flexible tube from the noise from this fan. Experiments were performed in the Acoustics Laboratory in the Department of Engineering at the University of Cambridge.

In the experiments relevant to this paper, the suction at (8) is gradually increased until the flexible tube just starts oscillating. With the tube oscillating in this way, high-speed video is recorded from 2 FASTCAM-ultima APX cameras (produced by Photron, https://photron.com) with a frame rate of 12 500 fps and a resolution of 512×256 pixels (greyscale). Our experiments require us to focus on a small flexible tube at reasonably close range. The Photron camera has an adaptor for Nikon lenses. We use a 50 mm lens combined with a 7 mm extension tube to allow us to focus on the tube and have it fill most of the frame. An aperture of f/2.8 is used.

The flexible tubes used are made out of rubber latex for which *E*=1 *MPa* and *ν*=0.5. The tube diameter is 6 mm, the wall thickness is 0.3 mm and the unstrained length is 19 mm. The tubes are held in an axially strained state, so the length of the tubes during the experiment is 25 mm.

### 3.2. Image processing

**Figure 2.**
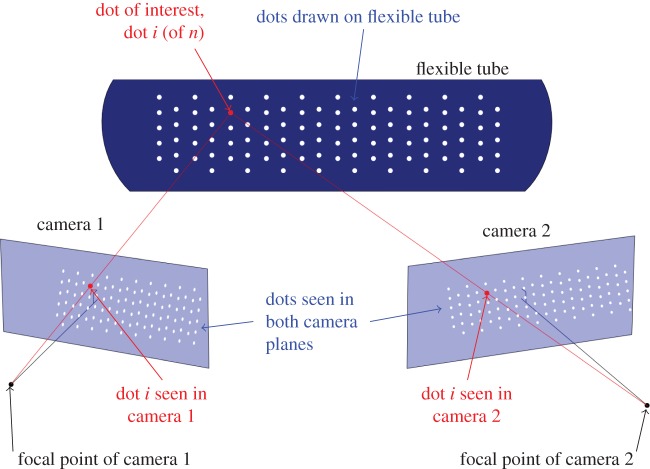
Schematic of the high-speed camera set-up.

The high-speed cameras record at 12 500 fps, and are triggered together, so that every *Δt*=80 μ*s*, two images of the flexible tube are taken. A schematic of the two cameras and the flexible tube is shown in figure 2. Dots are drawn on the flexible tube (shown in white in figure 2), which indicate a set of material points we aim to track over time in three dimensions.

It is possible to find the characteristics^2^ of two cameras such that if a point appears in simultaneous images from both cameras, the point’s position in three-dimensional space can be triangulated [24,25]. Calibration involves taking at least 3, and in general between 10 and 20, simultaneous images of a chequerboard pattern in various orientations. From this the position of the two cameras relative to each other, and their internal parameters, can be calculated. In this method, cameras are modelled as pinhole cameras, meaning that the focal length, pixel size and skew are the important internal parameters. In addition, it is possible to account for radial distortion of the image by the camera lens, and tangential distortion, which occurs when the image sensor is not perfectly perpendicular to the line of sight of the camera. This calibration is performed using the computer vision system toolbox of Matlab^®^ [26]. The images produced by these pinhole camera models is what is illustrated in figure 2.

To find the three-dimensional tracks of the material points, we must first find the locations of the dots within each image. We refer to these coordinate pairs as points. The dots on the tube surface are drawn in white, are spaced by approximately 1 mm and have a diameter of approximately 0.7 mm. We take the material points to be the centres of the dots, and we find them by first taking a two-dimensional convolution of the image with a ‘mexican hat’ function of the form

3.1 $$\frac{1}{\pi \sigma^{4}}\left(1-\frac{x^{2}+y^{2}}{2\sigma^{2}}\right)e^{-x^{2}+y^{2}/2\sigma^{2}},$$

where *σ* is the expected radius of the dot in the image. The convolution effectively smooths the image, removing any artefacts from drawing the dots, leaving peaks in the centroid of each dot. These peaks are then used as the locations of each point.

Hence at each time instance, we have two collections of points, representing the material points as seen from each camera. If there are *n* points on the tube, and *m* frames in our video, then in total we will have found the locations of 2*nm* points. To make use of this data, we need to identify each unique material point in each camera and over time.

To associate points across time for a single camera’s set of images, the points at *t* and *t*+*Δt* are compared, and if two points are within a certain distance of each other, then it is assumed that these represent the same point. This works because the frame rate (12 500 fps) is much larger than the frequency of the observed vibrations (approx. 500 *Hz*), so motions between frames are small.

A pair of corresponding points in the two camera images are illustrated in figure 2, but finding these pairings at each instant in time is more complex. First, we consider the line drawn from the focal point of camera 2 to dot *i* in the image, which we will call a ray. Anything on this ray in three-dimensional space will appear at the same highlighted location in camera 2. However, from camera 1, the ray will appear as a line. Therefore, if a point’s location is known in one camera image, then it must lie on a specific line in the other image. This line is known as an epipolar line [25]. Hence, for a pair of points, one in each camera image, to correspond to the same material point, they must each lie on the epipolar line of the other. However, because of the specific arrangement of the cameras and dots, this does not usually provide a unique set of pairs. The relative positioning of the cameras means that the epipolar lines are all approximately horizontal, and the dots drawn on the tube are arranged in horizontal rows, so multiple dots can be very close to a given epipolar line. To overcome this, we specify 10 corresponding point pairs between the two images (20 points in total), and these 10 pairs are then used to find the best fitting projective transformation from camera 1 to camera 2. Applying this transformation to the image from camera 1 places each point close to the corresponding point in the image from camera 2, allowing all the remaining point pairs to be found. This result is then checked for consistency with the epipolar line condition.

In addition to tracing material points over time as the self-excited oscillations occur, we must also find the locations of the material points on the tube when it is unstrained. This is necessary for the calculation of the kinematic variables used in the expression for energy of deformation (2.1). To achieve this, a single image is taken from each camera when the tube is held in its unstrained state. Pairing of points must then also be completed between the two images of the tube in its unstrained state and images from the high-speed video of self-excited oscillations. This pairing is done using the methods described in the previous paragraph.

Once point pairs are known over time, the camera calibration can be used to find three-dimensional point traces over time. The spatial resolution of this trace is limited by the size of the pixels in the high-speed video. This results in point traces with distinct jumps in position. These jumps are by no more than 0.1 mm in three-dimensional space, compared to variations in position of the order of 2 mm over the course of the self-excited oscillations. For this reason, we smooth the three-dimensional point traces by fitting functions of the form

3.2 $$\sum_{i=1}^{8}A_{i}\sin(\omega_{i}t),$$

to the three position components, where *A*_*i*_ and *ω*_*i*_ are chosen to fit the empirical data. These fits work well because the videos are of quasi-steady behaviour at the onset of oscillation, and the observed motions are close to sinusoidal. Eight terms have been found to be sufficient to match the experimental data. This smoothing is necessary in order for derivatives of the point traces to give meaningful results.

### 3.3. Kinematic calculations

**Figure 3.**
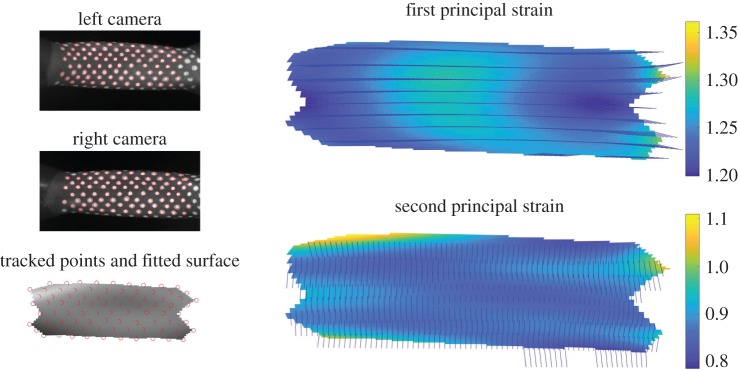
A typical image of the principal strains of the flexible tubes, shown along with the principal strain directions (illustrated with unit vectors). On the left the original high-speed camera images are shown with the tracked surface points, and a view of the three-dimensional triangulation with the surface fitted to them.

In figure 3, we show a single frame from the video in which the dots on the surface of the tube have been automatically detected, identified with the corresponding dots in the other video image and identified with the corresponding dots in a stereoscopic image of the unstrained tube (not shown). With this information, we can reconstruct the points in three dimensions and fit a surface to them. The process can be summarized as follows:

— Locate points in images of unstrained tube.
— Track points in high-speed video frames.
— Associate points between all images (as described in §3.2) and triangulate to have the position of each material point as a function of time, and its position on the unstrained tube.
— Assign a pair of coordinate values {*x*^*i*^} to each point for use as the convected surface coordinates.
— Fit a smoothing curve to every three-dimensional point as a function of time as described in §3.2.
— At a chosen time take the positions of all of the points and fit a polynomial surface such that we have position as a function of the surface coordinates {*x*^*i*^}. Repeat this for the unstrained surface.
— Take all possible first and second derivatives of the surface position with respect to {*x*^*i*^}. This is done analytically using the polynomial surface. Repeat this for the unstrained surface.

The surface fit and its derivatives are used to calculate all the kinematic properties of the undeformed and deformed surfaces.

**Figure 4.**
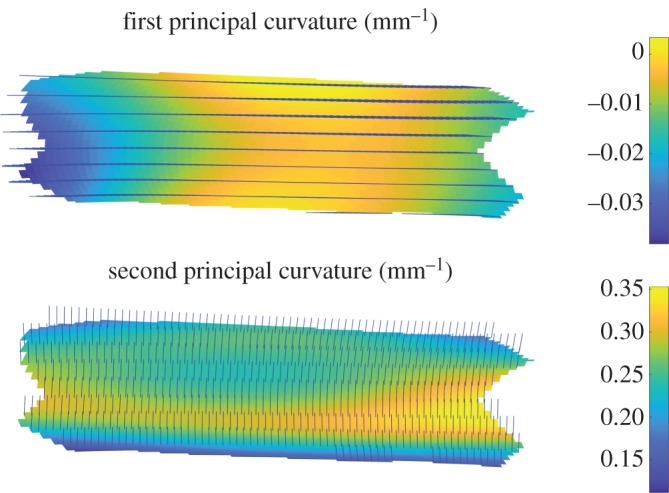
A typical image of the principal curvatures of the flexible tube, shown along with the principal curvature directions (illustrated with unit vectors).

In figure 3, we show a typical image of the principal strains of the flexible tube, i.e. the eigenvectors and values of E. An eigenvalue of 1 corresponds to no strain, a value less than 1 corresponds to compression and a value greater than 1 corresponds to tension. The eigenvectors give the direction in which the strain is occurring. We can see that the first principal strain is approximately aligned with the longitudinal direction and is tensile. This is due to the dominant pre-strain of the elastic tube. By contrast, the second principal strain, which is primarily in the azimuthal direction, is a mixture of compression and tension, and is generally closer to 1. In figure 4, we show the principal curvatures of the deformed surface, i.e. the eigenvectors and values of b. For a cylinder, which the tube is in its undeformed state, the principal curvatures would be 0 in the longitudinal direction and 1/*a*=0.33 mm^−1^ in the azimuthal direction. We see that, in the deformed tube, the curvatures are still closely aligned to the longitudinal and azimuthal directions, and can see that the squashing of the tube results in a slightly negative longitudinal curvature and a slight reduction in the azimuthal curvature towards the centre of the tube. But these effects are fairly small.

## 4. Calculation of bending and stretching energies

We now aim to gain more understanding of how the tube deforms. To do this, we will use a mixture of empirical and analytical techniques. More specifically, we can use the high-speed video reconstructions combined with the mathematical framework for shells already developed in §2.

### 4.1. Scaling analysis

We start by estimating the bending and stretching energies analytically, which requires us to estimate the values of tr(E^2^), tr(E)^2^, tr(H^2^) and tr(H)^2^.

If *α*_*i*_ are the eigenvalues of the linear function A, then $\mathrm{tr}{\left(A\right)}^{2}=\alpha_{1}^{2}+2\alpha_{1}\alpha_{2}+\alpha_{2}^{2}$ and $\mathrm{tr}(A^{2})=\alpha_{1}^{2}+\alpha_{2}^{2}$. We know that the eigenvalues of E are $\frac{1}{2}(\lambda_{i}^{2}-1)$ (λ_*i*_ are the principal strains) and that λ_1_∼λ, λ_2_∼1, where λ is the initial axial strain of the tube. This allows us to get an order of magnitude estimate for tr(E^2^) and tr(E)^2^ of (λ^2^−1)^2^/4. If we take λ=1.3,^3^ then we have tr(E^2^)∼tr(E)^2^∼0.1.

To understand bending in the tube, we consider the representation of H derived in §2, and given in (2.12). We can write the action of U(*Y*) as $U(Y)=\lambda_{1}(Y\cdot {\hat{W}}_{1}){\hat{W}}_{1}+\lambda_{2}(Y\cdot {\hat{W}}_{2}){\hat{W}}_{2}$, where ${\hat{W}}_{i}$ are the unit eigenvectors of U. Using the approximation λ_1_≈λ and λ_2_≈1, this becomes $U(Y)\approx \lambda (Y\cdot {\hat{W}}_{1}){\hat{W}}_{1}+(Y\cdot {\hat{W}}_{2}){\hat{W}}_{2}$. We can write B(*Y*) as $B(Y)=C(Y\cdot {\hat{E}}_{2}){\hat{E}}_{2}$, where ${\hat{E}}_{i}$ are the unit eigenvectors of B, and *C* is the principal curvature of the undeformed tube in the azimuthal direction. Combining these we have

4.1 $$\begin{array}{rl} UB(Y)-B(Y) & =U(CY\cdot {\hat{E}}_{2}{\hat{E}}_{2})-CY\cdot {\hat{E}}_{2}{\hat{E}}_{2}\\ & \approx CY\cdot {\hat{E}}_{2}(\lambda {\hat{E}}_{2}\cdot {\hat{W}}_{1}{\hat{W}}_{1}+{\hat{E}}_{2}\cdot {\hat{W}}_{2}{\hat{W}}_{2})-CY\cdot {\hat{E}}_{2}{\hat{E}}_{2}. \end{array}$$

We know from figure 3 that ${\hat{W}}_{1}$ and ${\hat{W}}_{2}$ are approximately aligned with the longitudinal and circumferential directions, so we can write ${\hat{E}}_{2}\cdot {\hat{W}}_{1}\approx 0$ and ${\hat{E}}_{2}\cdot {\hat{W}}_{2}\approx 1$. Using this, we obtain

4.2 $$UB(Y)-B(Y)\approx CY\cdot {\hat{E}}_{2}{\hat{W}}_{2}-CY\cdot {\hat{E}}_{2}{\hat{E}}_{2}\approx 0.$$

This is saying that because the directions of principal strain and principal curvature are approximately perpendicular, the influence of strain on the change of curvature is removed.

**Figure 5.**
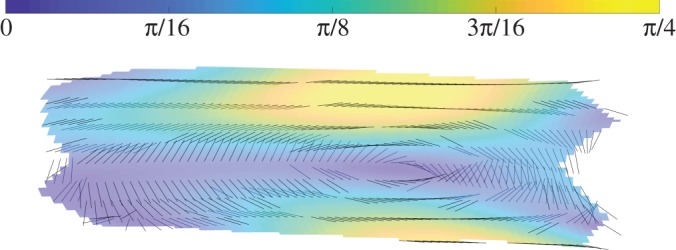
A visualization of the rotation R. The axis of rotation is shown along with the absolute value of the angle of rotation (in radians).

We are now in a position to consider the rotor *R*, because it is changes in this multivector over the surface of the shell that are responsible for the change of curvature. By *R* we represent the rotation, and as is shown in §2 it is characterized by the bivector $A=\theta \hat{A}$, whose magnitude gives the rotation angle in radians, and whose plane gives the plane of rotation. In figure 5, we visualize the angle and axis of rotation encoded in the rotation tensor R, corresponding to a typical deformation. The axes of rotation shown in figure 5 are primarily tangential to the surface, so the bivector *A* will be dominated by the components *e*_1_∧*e*_3_ and *e*_2_∧*e*_3_, with little rotation in the *e*_1_∧*e*_2_ plane, i.e. about the normal vector *e*_3_. Hence, we can write *A* as

4.3 $$A=\theta_{1}e^{1}\wedge e_{3}+\theta_{2}e^{2}\wedge e_{3}=\theta_{i}e^{i}\wedge e_{3}.$$

We have used the reciprocal frame {*e*^*i*^} instead of {*e*_*i*_} because it will allow us to use the property $\bar{F}(e^{i})=E^{i}$.^4^ We can extend the frames {*e*^*i*^} and {*e*_*i*_} to span $E^{3}$ by using the normal vector *e*_3_. Because *e*_3_ is a unit vector and perpendicular to all of *e*_*i*_,*e*^*i*^, we can also write *e*^3^=*e*_3_, and we have the frame {*e*_*a*_},*a*=1,2,3 and {*e*^*a*^}. Using this, we define the Christoffel coefficients $\gamma_{ib}^{a}=e^{a}\cdot \partial e_{b}/\partial x^{i}$, *i*=1,2;*a*,*b*=1,2,3. These also satisfy $e_{b}\cdot \partial e^{a}/\partial x^{i}=-\gamma_{ib}^{a}$.

Substituting *A* into the second part of the change of curvature tensor given in (2.12), using the fact that *e*_3_ is normal to *e*^1^ and *e*^2^, and $\gamma_{i3}^{3}=0$, we obtain

4.4 $$\begin{array}{rl} \bar{F}(e_{3}\cdot (Y\cdot \partial A)) & =Y^{i}\bar{F}(-\partial_{i}(\theta_{j})e^{j}+\theta_{j}\gamma_{ik}^{j}e^{k})\\ & =-Y^{i}\partial_{i}(\theta_{j})E^{j}+Y^{i}\theta_{j}\gamma_{ik}^{j}E^{k}. \end{array}$$

Therefore, given that the first part of H in (2.12) is zero, *E*_*i*_⋅H(*E*_*j*_)=H_*ij*_ is given by

4.5 $$H_{ij}=\partial_{j}\theta_{i}-\theta_{k}\gamma_{ji}^{k}.$$

We know that H is symmetric, so from this we see that our earlier assumption on the form of *A* must be joined by the condition ∂_*i*_*θ*_*j*_=∂_*j*_*θ*_*i*_ to produce consistent results.

**Figure 6.**
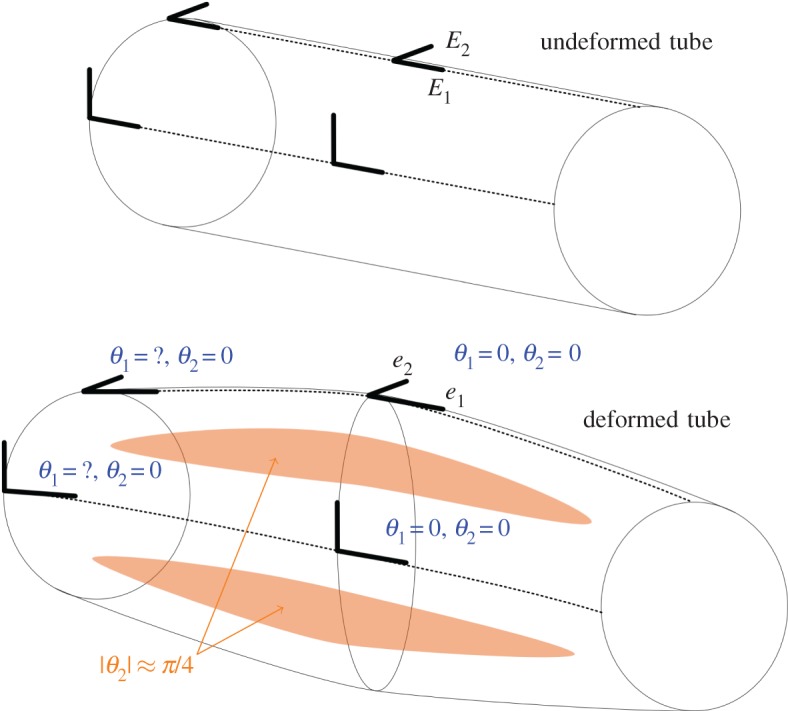
A schematic of the deformation of the flexible tube.

The frame {*E*_*i*_} can be chosen by us to be orthonormal. More specifically, we can align *E*_1_ with the longitudinal direction and *E*_2_ with the azimuthal direction on the unstrained cylindrical tube. From figure 3, we can see that under a typical deformation these basis vectors remain close to the axial and azimuthal directions. In figure 6, we give a schematic illustration of how *E*_*i*_ maps to *e*_*i*_. In figure 6, we have also labelled the values of *θ*_*i*_ where they are obvious. The regions where |*θ*_2_|≈*π*/4 can be seen empirically in figure 6. There are two unknown values of *θ*_1_ shown as question marks. We can estimate the largest value of these rotations by assuming a straight line from the clamped tube end and the centre of the tube when the tube collapses completely at the centre. In this case, $\theta_{1}∼\arctan(a/(l/2))\lambda$, where *a* is the tube radius and *l* is the tube length in its deformed state. The multiplication by λ is necessary because *θ*_1_ is the *e*^1^∧*e*_3_ component and *e*^1^ is shortened by a factor of λ compared to the unit vector *E*^1^. Up to angles of 30^°^, tan⁡θ is within 10% of *θ*, so we will take *θ*_1_∼*λa*/(*l*/2)=*a*/(*l*_0_/2) at the point in question, where *l*_0_ is the unstrained length of the tube. This, and the values of *θ*_*i*_ shown in figure 6, allow us to make the following estimates:

4.6 $$\left.\begin{array}{rl} & \partial_{1}\theta_{1}∼\frac{a}{l_{0}/2}\frac{1}{l_{0}/2}=\frac{4a}{l_{0}^{2}},\\ & \partial_{1}\theta_{2}=\partial_{2}\theta_{1}∼\frac{\pi /4}{l_{0}/2}=\frac{\pi }{2l_{0}}\\ \;\text{and}\; & \partial_{2}\theta_{2}∼\frac{\pi /4}{2\pi a/8}=\frac{1}{a}. \end{array}\right\}$$

Because of replacement of arctan with the identity function, our estimate for ∂_1_*θ*_1_ will be an overestimate when the tube is very short.

We can also estimate the values of the coefficients $\gamma_{jk}^{i}$ using figure 6,

4.7 $$\left.\begin{array}{rl} \partial_{1}e_{1} & ∼\frac{a}{{\left(l_{0}/2\right)}^{2}}e_{3},\\ \partial_{2}e_{1} & =\partial_{1}e_{2}∼0\\ \;\text{and}\;\partial_{2}e_{2} & ∼\frac{1}{a}e_{3}. \end{array}\right\}$$

From this we see that the changes in the basis vectors are primarily in the *e*_3_ direction, meaning that they do not contribute to H_*ij*_.

If we take *l*_0_ to be much larger than *a*, then the dominant term in H_*ij*_ will be 1/*a*, but even if *l*_0_ and *a* are of a similar order of magnitude, all of the H_*ij*_ terms will be of the order 1/*a*. Hence, we expect tr(H^2^) and tr(H)^2^ will scale as (1/*a*)^2^=(1/3 mm)^2^=0.1 mm^−2^.

Given these scalings for tr(E^2^), tr(E)^2^, tr(H^2^) and tr(H)^2^, and the values *E*=1 *MPa*, *ν*=0.5 and *h*=0.3 mm, we can obtain scalings for the bending and stretching energy given in (2.1):

4.8 $$\left.\begin{array}{rl} \text{stretching energy} & ∼0.02\;{N\;\mathrm{mm}}^{-1}\\ \;\text{and}\;\text{bending energy} & ∼1.5\times 10^{-4}\;{N\;\mathrm{mm}}^{-1}. \end{array}\right\}$$

This indicates that given the kind of deformation we have observed in our Starling resistors at onset, i.e. where the strain energy is dominated by the effects of pre-strain, the axis aligned with the largest strain remains close to perpendicular to the axis aligned with the largest curvature, rotations are mostly about axes tangential to the shell, and changes in the rotation scale with the change of rotation about the longitudinal axis in the azimuthal direction, stretching energy will dominate bending energy. Moreover, this result remains valid even when the tube length gets close to the tube diameter. This is significant for our considerations of the lung, because the length to diameter ratio of tubes in the lung typically varies from 1 to 6 [27].

### 4.2. Direct calculation from data

**Figure 7.**
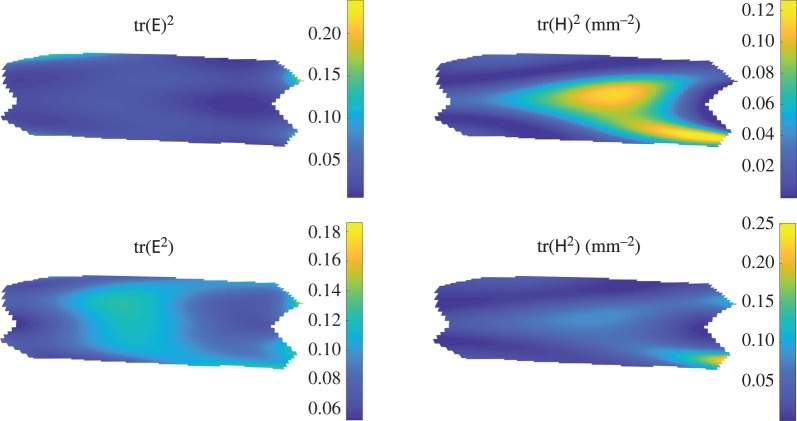
Values of the kinematic variables tr(E^2^), tr(E)^2^, tr(H^2^) and tr(H)^2^.

**Figure 8.**
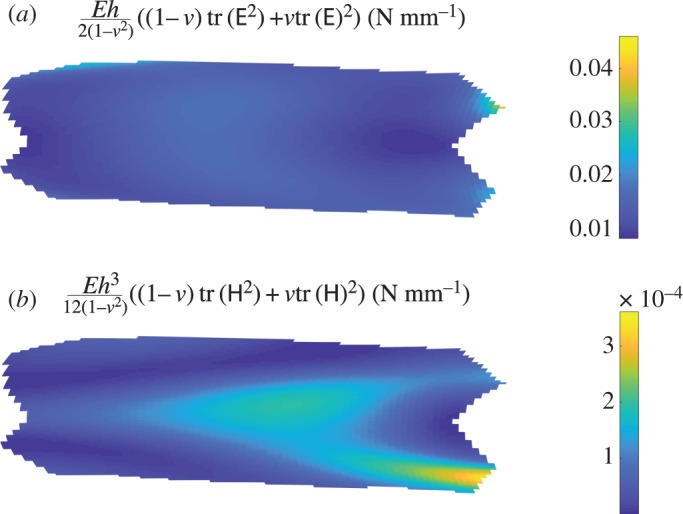
The stretching energy (*a*) and bending energy (*b*) associated with the deformation of the flexible tube.

We can use the high-speed video data to calculate tr(E^2^), tr(E)^2^, tr(H^2^) and tr(H)^2^ from (2.1). Typical plots are shown in figure 7, from which we see that in the units chosen these have similar orders of magnitude. We also see that the scalings obtained in the previous section agree with these plots well, providing support for the assumptions made. We can also calculate the bending and stretching energy, and this is shown in figure 8. This agrees very well with the scaling values of the previous section, again supporting our conclusions.

## 5. Conclusion

We have developed a new method of representing the change of curvature tensor using rotors (see (2.12)), increasing our understanding of bending in shells. We have used this representation to explain results from stereographic imaging of Starling resistors that demonstrate that the bending energy in these deformations is approximately 2 orders of magnitude lower than the stretching energy. We have been able to show that this relies on the fact that the strain energy is dominated by the effects of pre-strain, the axis aligned with the largest strain remains close to perpendicular to the axis aligned with the largest curvature, rotations are mostly about axes tangential to the shell and changes in the rotation scale with the change of rotation about the longitudinal axis in the azimuthal direction. Further to this, our scaling analysis remains valid even when the tube length gets close to the tube diameter. This is of significance to our work in understanding wheezing, because the length to diameter ratio of tubes in the lung typically varies from 1 to 6. Hence we have provided a scaling analysis, confirmed by experiment, that allows us to say that bending energy is dominated by stretching energy during self-excited oscillations in the airways of the lung. This should allow the use of membrane theory to model the tube, which reduces the order of the equations of motion from 4 to 2.

## Footnotes

1 The coordinate system {*X*^*i*^} must be chosen such that this is the case, and it may be necessary to use several overlapping coordinate systems to achieve this.

2 The characteristics of an imaginary pinhole camera that we replace the real camera with to allow us to use methods from projective geometry to perform the triangulation.

3 The strained length of the tube is *l*=25 mm and the unstrained length is *l*_0_=19 mm, giving λ=*l*/*l*_0_≈1.3.

4 The coordinate system is convected, so *e*_*i*_=F(*E*_*i*_), and as always $e^{i}\cdot e_{j}=E^{i}\cdot E_{j}=\delta_{j}^{i}$. Hence $\delta_{j}^{i}=e^{i}\cdot F(E_{j})=\bar{F}(e^{i})\cdot E_{j}$, from which it is clear that $\bar{F}(e^{i})=E^{i}$.

## Data accessibility

Data for this paper can be accessed at University of Cambridge Repository: https://doi.org/10.17863/CAM.10363 [28].

## References

[1] Heil M, Jensen OE 2003 Flows in deformable tubes and channels. In *Flow past highly compliant boundaries and in collapsible tubes* (eds PW Carpenter, TJ Pedley), pp. 15–49. Dordrecht, The Netherlands: Kluwer Academic Publishers.

[2] Bertram CD 2003 Experimental studies of collapsible tubes. In *Flow past highly compliant boundaries and in collapsible tubes* (eds PW Carpenter, TJ Pedley), pp. 51–65. Dordrecht, The Netherlands: Kluwer Academic Publishers.

[3] Grotberg JB, Jensen OE 2004 Biofluid mechanics in flexible tubes. *Annu. Rev. Fluid Mech.* 36, 121–147. (doi:10.1146/annurev.fluid.36.050802.121918)

[4] Heil M, Hazel AL 2011 Fluid-structure interaction in internal physiological flows. *Annu. Rev. Fluid Mech.* 43, 141–162. (doi:10.1146/annurev-fluid-122109-160703)

[5] Kisilova N, Hamadiche M, Gad-El-Hak M 2012 Mathematical models of biofluid flows in compliant ducts. *Arch. Mech.* 64, 65–94.

[6] Jensen OE 2013 Instabilities of flows through deformable tubes and channels. In *Mechanics Down Under—Proc. of the 22nd Int. Congress of Theoretical and Applied Mechanics, ICTAM 2008*, *Adelaide, Australia* (eds J Denier, M Finn), pp. 101–116.

[7] Grotberg JB, Davis SH 1980 Fluid-dynamic flapping of a collapsible channel: sound generation and flow limitation. *J. Biomech.* 13, 219–230. (doi:10.1016/0021-9290(80)90365-6) 7372685 10.1016/0021-9290(80)90365-6

[8] Forgacs P 1978 *Lung sounds*. London, UK: Baillière Tindall.

[9] Bohadana AB, Izbicki G, Kraman SS 2014 Fundamentals of lung auscultation. *N. Engl. J. Med.* 370, 744–751. (doi:10.1056/NEJMra1302901) 24552321 10.1056/NEJMra1302901

[10] Whittaker RJ, Heil M, Jensen OE, Waters SL 2010 A rational derivation of a tube law from shell theory. *Q. J. Mech. Appl. Math.* 63, 465–496. (doi:10.1093/qjmam/hbq020)

[11] Shabtai-Musih Y, Grotberg JB, Gavriely N 1992 Spectral content of forced expiratory wheezes during air, He, and SF_6_ breathing in normal humans. *J. Appl. Physiol.* 72, 629–635. 1559941 10.1152/jappl.1992.72.2.629

[12] Leissa AW 1973 Vibration of shells. Technical Report NASA SP-288. Washington, DC: National Aeronautics and Space Administration.

[13] Naghdi PM 1972 The theory of shells and plates. In *Handb. der Phys. (Encyclopedia Physics), Band (Volume) VIa/2, Festkorpermechanik II (Mechanics Solids II)* (eds S Flugge, C Truesdell), pp. 425–640. Berlin, Germany: Springer.

[14] Ciarlet PG Jr 2005 An introduction to differential geometry with applications to elasticity. *J. Elast.* 78–79, 1–215. (doi:10.1007/s10659-005-4738-8)

[15] Antman SS 2005 *Nonlinear problems of elasticity*. 2nd edn Berlin, Germany: Springer.

[16] Lacarbonara W 2012 The nonlinear theory of plates. In *Nonlinear structural mechanics* (ed. W Lacarbonara), pp. 497–592. Boston, MA: Springer.

[17] Koiter WT 1966 On the nonlinear theory of thin elastic shells. *Koninklijke Nederlandse Akademie van Wetenschappen* 69B, 1–54.

[18] Gregory AL, Lasenby J, Agarwal A 2017 The elastic theory of shells using geometric algebra. *R. Soc. open sci.* 4, 170065 (doi:10.1098/rsos.170065) 28405404 10.1098/rsos.170065 PMC5383861

[19] Doran CJL, Lasenby AN 2003 *Geometric algebra for physicists*. Cambridge, UK: Cambridge University Press.

[20] McRobie FA, Lasenby J 1999 Simo-Vu Quoc rods using Clifford algebra. *Int. J. Numer. Methods Eng.* 45, 377–398. (doi:10.1002/(SICI)1097-0207(19990610)45:4<377::AID-NME586>3.0.CO;2-P)

[21] Simo JC, Vu-Quoc L 1988 On the dynamics in space of rods undergoing large motions—a geometrically exact approach. *Comput. Methods Appl. Mech. Eng.* 66, 125–161. (doi:10.1016/0045-7825(88)90073-4)

[22] Lasenby A, Doran C, Gull S 1998 Gravity, gauge theories and geometric algebra. *Phil. Trans. R. Soc. Lond. A* 356, 487–582. (doi:10.1098/rsta.1998.0178)

[23] Gregory AL, Sinayoko S, Agarwal A, Lasenby J 2015 An acoustic space-time and the Lorentz transformation in aeroacoustics. *Int. J. Aeroacoust.* 14, 977–1003. (doi:10.1260/1475-472X.14.7.977)

[24] Dorst L, Fontijne D, Mann S 2009 *Geometric algebra for computer science: an object-oriented approach to geometry*, 2nd edn Los Altos, CA: Morgan Kaufmann.

[25] Hartley R 2004 *Multiple view geometry in computer vision*, 2nd edn Cambridge, UK: Cambridge University Press.

[26] Matlab. 2017 *version 9.2.0 (R2017a)*. Natick, MA: The Mathworks Inc.

[27] Horsefield K, Cumming G 1968 Morphology of the bronchial tree in man. *J. Appl. Physiol.* 24, 373–383. 5640724 10.1152/jappl.1968.24.3.373

[28] Gregory AL, Agarwal A, Lasenby J 2017 Data from: Using geometric algebra to represent curvature in shell theory with applications to Starling resistors. University of Cambridge Repository (doi:10.17863/CAM.10363) 10.1098/rsos.171212 PMC5717681 29291106

